# Effects of microtubule length and crowding on active microtubule network organization

**DOI:** 10.1016/j.isci.2023.106063

**Published:** 2023-01-27

**Authors:** Wei-Xiang Chew, Gil Henkin, François Nédélec, Thomas Surrey

**Affiliations:** 1 Centre for Genomic Regulation (CRG), Barcelona Institute of Science and Technology (BIST), Dr Aiguader 88, 08003 Barcelona, Spain; 2 Sainsbury Laboratory, University of Cambridge, 47 Bateman Street, Cambridge CB2 1LR, UK; 3 ICREA, Passeig de Lluis Companys 23, 08010 Barcelona, Spain

**Keywords:** Biological sciences, Cell biology, Functional aspects of cell biology, Biophysics

## Abstract

Active filament networks can organize into various dynamic architectures driven by cross-linking motors. Densities and kinetic properties of motors and microtubules have been shown previously to determine active microtubule network self-organization, but the effects of other control parameters are less understood. Using computer simulations, we study here how microtubule lengths and crowding effects determine active network architecture and dynamics. We find that attractive interactions mimicking crowding effects or long microtubules both promote the formation of extensile nematic networks instead of asters. When microtubules are very long and the network is highly connected, a new isotropically motile network state resembling a “gliding mesh” is predicted. Using *in vitro* reconstitutions, we confirm the existence of this gliding mesh experimentally. These results provide a better understanding of how active microtubule network organization can be controlled, with implications for cell biology and active materials in general.

## Highlights

• Computer simulations provide insight into mechanisms of active network generation
• Microtubule length and crowding favor nematic microtubule/motor network formation
• Simulations and experiments reveal a new isotropic active microtubule network state

## Graphical abstract

**Figure:**
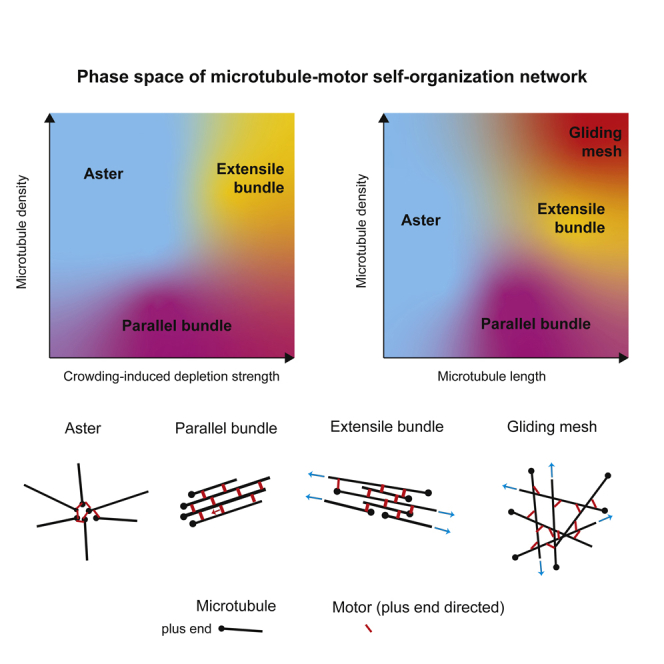

## Introduction

Active filament networks, driven out of equilibrium by ATP-consuming cross-linking motor proteins, have the capacity to adopt different dynamic organizations. In living cells, they play important roles in various processes such as spindle assembly,^1^ cytoplasmic streaming,^2^ and cell shape control.^3^ Biochemical reconstitutions with purified proteins *in vitro* have made important contributions to our understanding of active network organization and dynamics.^4^^,^^5^^,^^6^^,^^7^ Networks formed from microtubules and motors can display various architectures, among which radially polar networks (asters)^8^^,^^9^^,^^10^^,^^11^^,^^12^ and networks of extensile mixed-polarity bundles have been most frequently studied.^13^^,^^14^^,^^15^^,^^16^ The molecular parameters that determine these different collective behaviors remain, however, incompletely understood.

Microtubules are dynamic, structurally polar filaments with distinct “plus” and “minus” ends, composed of tubulin subunits arranged into a tubular structure. Early *in vitro* experiments with artificially oligomerized plus-end-directed kinesin-1 and microtubules growing in solution showed the formation of contractile networks, eventually forming asters with a radially polar microtubule arrangement.^8^^,^^11^ Computer simulations demonstrated that aster formation depends on the motors' ability to remain bound upon reaching microtubule ends, allowing the ends to be brought together. Similar networks were later observed in the presence of the natural microtubule cross-linking motors kinesin-5 and kinesin-14, which walk toward the plus or minus ends, respectively, and form asters with opposite polarity.^10^^,^^15^^,^^17^

Extensile networks of mixed-polarity microtubule bundles were first reconstituted *in vitro* using short pre-polymerized microtubules and artificially oligomerized kinesin-1 motors in the additional presence of a crowding agent causing microtubule bundling by depletion forces.^13^ Microtubules within the bundles were aligned with their closest neighbors forming a nematic network. Because microtubule polarity was mixed within the nematic bundles, cross-linking motors drove their extension. At lower concentrations of crowding agents, or a reduced density of static microtubules, these networks were shown to become contractile,forming asters.^14^ Dynamic microtubules growing in solution in the absence of a crowding agent can also be organized by motors into extensile nematic networks, provided the tubulin concentration is high enough to promote fast microtubule growth and generate high microtubule densities. Computer simulations showed that fast microtubule growth relative to motor speed and high microtubule densities favor extensile nematic network formation, whereas slower microtubule growth or faster motor speed facilitates the accumulation of the motor at microtubule ends, causing network contraction into asters.^15^

Altogether, qualitatively similar networks have been observed both with and without crowding agents and with both shorter and longer microtubules. While theoretical models and computer simulations have helped to explain the effects of certain control parameters in self-organized microtubule-motor networks (i.e., microtubule growth rate, microtubule density, motor speed, motor density, motor composition),^15^^,^^18^^,^^19^^,^^20^ other parameters that appeared determinant in *in vitro* studies remain unexplored. Particularly, a theoretical exploration of the effects of crowding-induced depletion forces and microtubule length has not been performed yet, hindering a consolidated understanding of active microtubule network organization.

Here we explore the effects of these control parameters on simulated active networks composed of microtubules and motors. We find that short-range attractive forces between microtubules which mimic the depletion interaction, promote bundling, preventing aster formation, and particularly at high microtubule densities, generate networks of extensile bundles. In the absence of such short-range attractive forces, shorter microtubules promote the formation of asters, whereas long microtubules promote the formation of extensile bundles, or when the network connectivity is high, the formation of an isotropic network with motile microtubules resembling a “gliding mesh”, a new network state whose existence we also demonstrate experimentally. Our simulations explain how the studied control parameters determine which types of microtubule links the motors form, which in turn determines active network organization.

## Results

**Figure 1:**
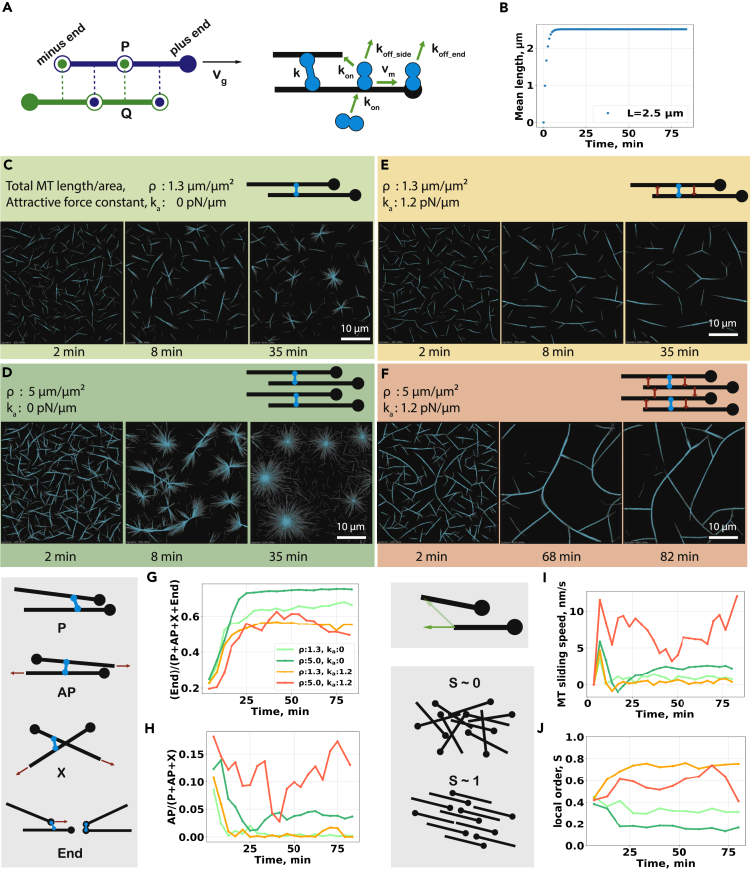
Simulations of active microtubule networks in the presence of an attractive depletion force (A) Elements of the microtubule/motor simulation. (Left) Microtubule filaments are discretized into points separated by an equal distance, allowing a filament to bend but not stretch. Each point is subjected to forces from bending elasticity and interaction with the cross-linker and other filaments. For the steric interaction, we consider all constitutive points P of a filament and project them onto the segments of other filaments. The force is orthogonal to the opposite filament at the projection point Q. An opposite force is applied to the first filament in P. The attractive force is implemented in the same way. Microtubules grow at a constant rate only at the plus end. (Right) Motors have two microtubule-binding units. A free motor unit binds to a filament with a binding rate *k*_*on*_*.* Motors that connect two filaments form a Hookean cross-link. A bound motor unit moves with a speed that is linearly dependent on the force, as defined by the stall force and the unloaded speed *v*_*m*_. Depending on its position along the filament, a motor can detach at a rate of *k*_*off_side*_ or *k*_*off_end*_. (B) Time course of the microtubules’ mean length with final length of 2.5 μm. (C and D) Time course of microtubule (gray) organization at (C) low total microtubule length per area (1.3 μm/μm^2^) and (D) high total length per area (5 μm/μm^2^) in the presence of KIF11 motor (cyan) without attractive interfilament depletion force. (E and F) Time course of active microtubule organization in the presence of attractive depletion force *k*_*a*_ (1.2 pN/μm) at (E) low and high (F) total microtubule length per area. (G-J) Motor cross-links are categorized depending on the angle between the microtubules and whether they occur near the microtubule minus ends. P links connect parallel microtubules where the internal angle is smaller than 60°. AP links connect antiparallel microtubules at an angle between 120° and 180°. X links connect microtubule sides when these microtubules form an angle from 60° to 120°. End links connect one or both microtubule ends. Time series of (G) the fraction of end-bound motors relative to all types of cross-links, (End links)/(P + AP + X + End) (see STAR Methods), (H) the fraction of side-bound motors that form antiparallel links relative to all non-end links, AP/(P + AP + X), (I) the mobility of microtubule minus ends along the filament axis, and (J) the local nematic order parameter calculated with a sampling window size of 10 μm. The KIF11 motor-to-microtubule ratio is 16. The simulation extends for 80 min in a box with dimensions 40 μm × 40 μm × 0.2 μm with periodic boundary conditions in x and y dimensions. See also Video S1 and Figure S1.

We simulated active networks consisting of microtubules and microtubule cross-linking motors using Cytosim (see key resources table). Microtubules and motors were modeled essentially as described earlier^16^^,^^21^ (see Figure 1A and STAR Methods). Microtubules grew in a thin and flat three-dimensional geometry from a fixed number of nucleators by plus-end elongation and repelled each other via soft-core interactions. Motors with the ability to bind two different microtubules could organize them into active networks. In this work, the motor properties mimicked those of the human spindle motor KIF11, a plus-end-directed microtubule cross-linking motor.

### Effects of a short-range attractive force between microtubules on active network organization

We first studied the effect of a crowding agent-induced depletion force between microtubules, in systems with different microtubule densities. We approximate the effects of the depletion force by a short-range attraction between microtubules (see STAR Methods). These forces promote the formation of bundles in which adjacent microtubules are free to slide longitudinally relative to each other and thus mimic the effects induced by crowding agents in experimental active networks.^13^ Microtubules grew with a speed that was initially equal to the motor speed, and then growth slowed down and finally stopped after ∼8 min when microtubules reached an average length of 2.5 μm (Figure 1B). Motors remained bound at microtubule ends for an average of 5 s, allowing them to form asters within 35 min in the absence of an attractive depletion force (Figures 1C and 1D), as observed previously in experiments and simulations.^15^^,^^16^ Whereas microtubules contracted locally into small disconnected asters at a lower microtubule density (Figure 1C), at a higher microtubule density, the network was initially highly interconnected, but subsequently broke down into smaller clusters (Figure S1) and finally contracted into individual asters (Figure 1D) that contain a large number of microtubule end-bound motors (Figure 1G).

Under these conditions, introducing an attractive force between the microtubules suppressed aster formation and instead caused the formation of microtubule bundles (Figures 1E and 1F), similar to experiments with a crowding agent.^14^ Isolated parallel microtubule bundles or parallel bundles connected by their plus ends formed at a lower microtubule density (Figure 1E) in which individual microtubules were fairly static, as indicated by a low microtubule sliding speed (Figure 1I) and a small number of motor links connecting antiparallel microtubules (Figure 1H). At a higher microtubule density, bundles extended and collapsed onto each other allowing them to fuse and continue extending (Figure 1F, Video S1). The networks displayed fast microtubule sliding with a fluctuating average speed (Figure 1I) and a large but also fluctuating number of motor links between antiparallel microtubules (Figure 1H). The behavior of the extensile bundles is also characterized by a relatively high, fluctuating local nematic order parameter (Figure 1J). The extending, bending, and recombining bundles in these simulations are reminiscent of active extensile networks observed experimentally in the presence of crowding agents.^13^

Video S1. Time course of simulated self-organizing networks driven by a KIF11-like motor (cyan) without attractive bundling force that form small asters (top left, density: 1.3 μm/μm^2^, k_a_: 0 pN/μm) and large asters (top right, density: 5 μm/μm^2^, k_a_: 0 pN/μm) and with attractive bundling force that form parallel bundles (bottom left, density: 1.3 μm/μm^2^, k_a_: 1.2 pN/μm) and extensile bundles (bottom right, density: 5 μm/μm^2^, k_a_: 1.2 pN/μm)

**Figure 2:**
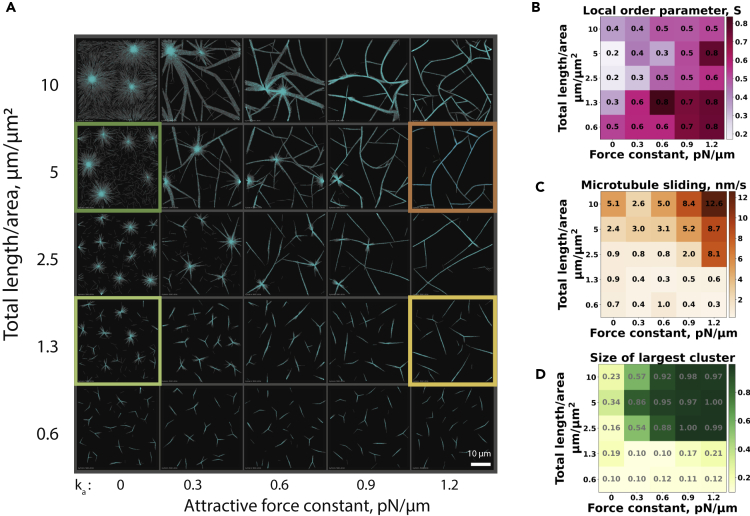
Organizational phase space and active network characteristics at various attractive forces (A) Organizational phase space of the microtubule network at various combinations of total microtubule length per area and attractive force strength. The four colored squares correspond to conditions in Figures 1C–1F. All boxes have the same size. (B) Local nematic order measured for each simulation shown in (A) at 80 min. (C) Calculated microtubule mobility for each simulation shown in (A). (D) Size of the largest cluster calculated for all simulations shown in (A) (normalized by total number of microtubules). For B, C, and D, the color scales are linear as indicated (right). The ranges correspond to the minimum and maximum values in the entire set.

**Figure 3:**
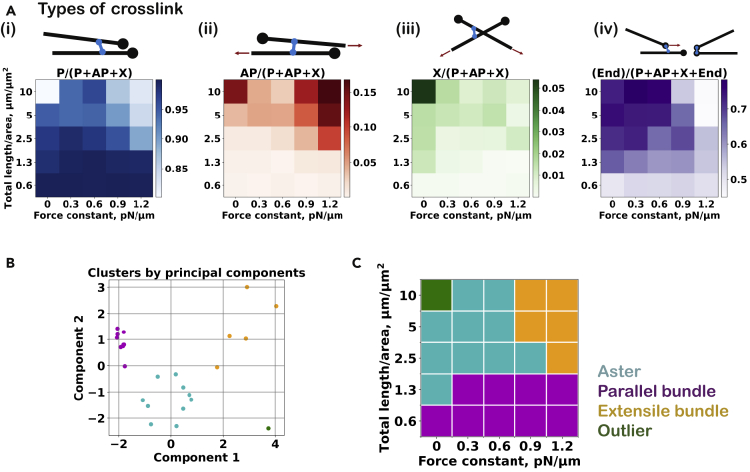
Topology of motor crosslinks and PCA-cluster analysis at various attractive forces (A) The fraction of each link type is represented using different color shades for different simulations, varying microtubule density, and attractive force strength: (i) P links, (ii) AP links, (iii) X links, and (iv) end links. The color scales are linear, corresponding to the minimum and maximum observed in the entire dataset. Original numerical values of the same data are reported in Figure S5A. (B) Scatterplot of the 25 microtubule organizations in the subspace of the two greatest principal components (see the breakdown of each component in Figure S2A). The four clusters identified via the K-means analysis are indicated by different colors: cyan as aster, magenta as parallel bundle, orange as extensile bundles, and green as outlier. (C) The corresponding states in the density-attractive force constant plot (see the characteristic descriptors of each state in Figure S2B).

Next, we explored a larger part of the organizational phase space to elucidate more systematically the combined effects of varying both the strength of the short-range attractive force and the microtubule density (Figure 2A). Extracting the local nematic order parameter from the simulated end states showed that generally, increasing the attractive force leads to more local nematic order (Figure 2B). The lowest degree of local nematic order was observed when most microtubules were incorporated into asters, whereas bundling increased the order parameter. For the highest attraction forces and the densest systems, the average microtubule sliding speed was the highest (Figure 2C), correlating with the highest network connectivity (Figure 2D) and the largest fraction of motor cross-links engaged in antiparallel microtubule contacts (Figure 3Aii). These observations demonstrate that microtubules fail to polarity-sort in the extensile bundle regime because extending bundles keep recombining with other bundles, which keeps their polarities mixed.

To quantitatively localize the three network states observed (aster, extensile bundle, and parallel bundle) in the organizational phase space, we performed a clustering procedure (see STAR Methods) (Figure 3B) using the two principal components of the seven network descriptors: the scalar nematic order parameter (Figure 2B), the microtubule speed (Figure 2C), the size of the largest cluster (Figure 2D), and the fractions of parallel links, antiparallel links, X cross-links, and end links, as defined in Figure 3Ai–Aiv. The identified clusters (Figure 3C) correspond well to the observed network states, and the network descriptors vary in a characteristic manner between the states: (i) the aster-forming state is characterized by a low order parameter and a large fraction of end links, (ii) the parallel bundle state displays a high nematic order parameter, a large fraction of parallel links, and a small cluster size, and (iii) the extensile bundle state shows a high microtubule sliding speed, a large fraction of antiparallel links, and a large cluster size. The network organization at the highest density without an attractive force (a focused aster with radially aligned microtubules) appears to be an outlier that shares characteristics of (i) and (iii) as can be seen in the principal component plot (bottom right of Figure 3B).

Together, these simulations show that both the increased attractive force between microtubules and the microtubule density promote a network of extensile microtubule bundles.

### Effects of microtubule length on active network organization

**Figure 4:**
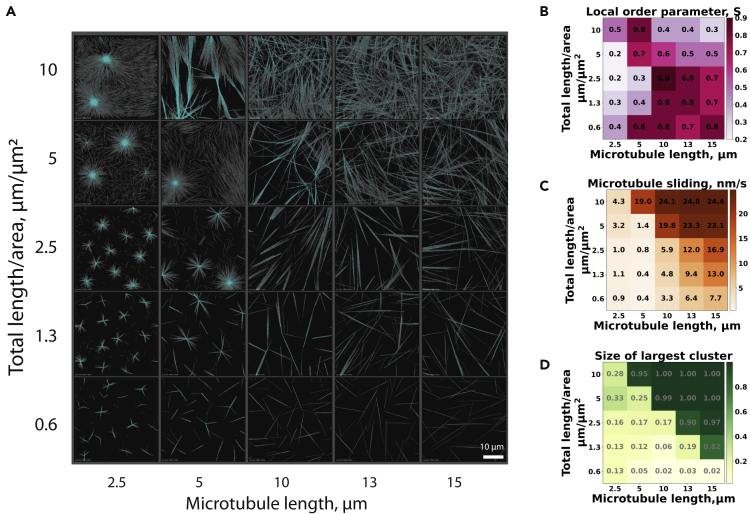
Organizational phase space and active network characteristics at various microtubule lengths (A) Organization of microtubule networks depending on average microtubule length and microtubule density. The KIF11 motor-to-microtubule ratio is 16. The simulation was performed for 60 min in a box of dimensions Lx μm × Ly μm × 0.2 μm with periodic boundary conditions in x and y dimensions, where Lx = Ly = 16 × (microtubule length). The microtubule length was varied from 2.5 to 15 μm (Figure S3B). For visualization purposes, only a part of the entire simulation space is shown with a constant area of 40 μm × 40 μm. The entire simulation spaces are shown in Figure S3A. (B) Local nematic order measured in each simulation shown in (A) at 60 min. (C) Calculated microtubule mobility for all simulations shown in (A). (D) Size of the largest cluster calculated for all simulations shown in (A) (normalized by total number of microtubules). For B, C, and D, the color scales are linear, corresponding to the minimum and maximum values observed in the dataset. See also Videos S2 and S3.

Next, we studied the effect of microtubule length on network organization. This was done in the absence of a short-range attractive force between microtubules to mimic *in vitro* experiments in which microtubule self-organization was studied by varying the tubulin concentration in the absence of crowding agents.^15^^,^^16^ In our simulations, we systematically varied the total microtubule density, defined as the total length of all microtubules per unit area, and the mean microtubule length that is reached toward the end of simulations (Figure 1B). Increasing the microtubule length from 2.5 μm to 10 μm at an intermediate microtubule density (2.5 μm total microtubule length per μm^2^) prevented individual aster formation and led to the formation of a polarity-sorted network containing polar bundles (Figure 4A, Video S2). Concomitantly, the local order parameter increased from 0.2 to 0.9 (Figure 4B), and the average microtubule sliding speed increased from 1 nm/s to 6 nm/s (Figure 4C). Further increasing the microtubule length caused a slight reduction of the nematic order to 0.7 but a continued increase of the microtubule sliding speed up to 17 nm/s and an increase of the cluster size toward the percolation threshold (Figure 4D), suggesting a transition into the extensile bundle state.

Video S2. Time course of the organization of microtubules (gray) and KIF11 motors (cyan) in the absence of an attractive bundling force that form asters (left, L: 2.5 μm), parallel bundles (middle, L: 10 μm), and a network of extensile bundles (right, L: 15 μm) Total microtubule length per area is 2.5 μm/μm^2^.

At higher microtubule densities (10 μm total microtubule length per μm^2^), the same trend was observed for simulations with increasing microtubule length (Figure 4A, Video S3A). However, the network with the maximum order parameter, consisting of extensile bundles, was already obtained at a shorter microtubule length of 5 μm, followed by a decrease in the order parameter when microtubule length increased further (Figure 4B). The microtubule sliding speed increased further with increasing microtubule length approaching 25 nm/s (close to the speed of the motors at 30 nm/s) for the longest microtubules (Figure 4C). Concomitantly, network connectivity increased sharply, reaching percolation at 10 μm microtubule length (Figure 4D). This state was also characterized by a relatively high fraction of X links. This seems to indicate a transition to a different type of network organization that shows fast microtubule sliding in a rather isotropic, highly percolated microtubule network.

Video S3A. Time course of the organization of microtubules (gray) and KIF11 motors (cyan) in the absence of an attractive bundling force that form asters (left, L: 2.5 μm), extensile bundles (middle, L: 5 μm), and a ‘gliding mesh’ organization (right, L: 15 μm)

**Figure 5:**
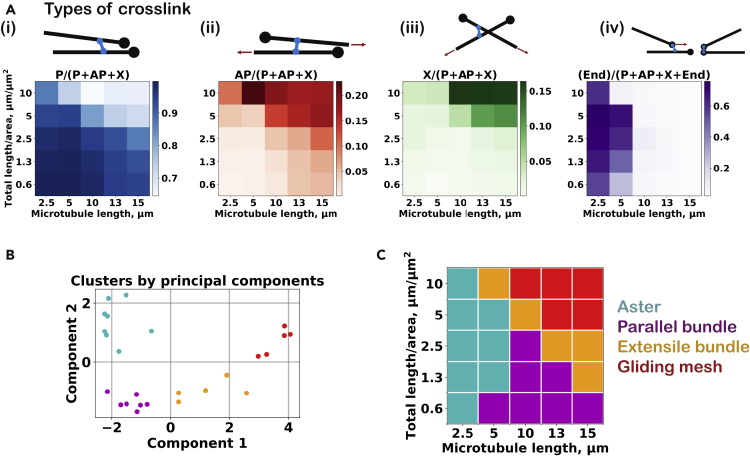
Topology of motor crosslinks and PCA-cluster analysis of networks formed at various microtubule lengths (A) Fractions of the different types of motor cross-links at each microtubule density and microtubule lengths: (i) P links, (ii) AP links, (iii) X links, and (iv) end links. The color scales are linear, corresponding to the minimum and maximum values in the entire dataset. Numerical values are reported in Figure S5B. (B) Scatterplot of the 25 microtubule organizations in the subspace of the two greatest principal components (see the breakdown of each component in Figure S2C). The four clusters identified via the K-means analysis are: aster (cyan), parallel bundle (magenta), extensile bundles (orange), and gliding mesh (red). (C) The corresponding states in the density-length plot (see the characteristic descriptors of each state in Figure S2D).

Having observed four different network organizations, we again performed a clustering analysis based on the seven network descriptors (Figures 4B–4D and 5A) to better identify the localization of each distinct network type in the microtubule length/density phase space. The identified clusters again corresponded well to the observed network states (Figures 5B and 5C), and the analysis was fairly robust against leaving out single descriptors for the analysis (Figure S4). Contractile networks forming asters were observed when microtubules were short. This state was characterized by low nematic order and slow microtubule motility. When microtubules are short, the motors can efficiently accumulate at microtubule ends, as indicated by a high fraction of end links. Parallel bundles formed when microtubules were long at low-to-intermediate microtubule densities. They were characterized by a high nematic order, a large fraction of parallel links, and very slow microtubule motility.

For the longest microtubules and the highest densities, the networks were percolated (cluster size of 1 in Figure 4D) with many cross-links between nonaligned microtubules (many X links in Figure 5Aiii). This network remains fairly isotropic (low order parameter in Figure 4B) because the high network connectivity hinders microtubule alignment or end gathering. Instead, the microtubules translocate through the network roughly in the direction of their longitudinal axis (Video S3B). The network remains isotropic since microtubules move in all directions equally, and because the apparent crosspoints of microtubules do not move much, the mesh itself also appears not to move much, although fluctuations reveal that it is by no means static. We refer to this network state as a “gliding mesh” in analogy to “gliding assays” in which surface-bound motors propel microtubules in all directions along the glass surface.

Video S3B. Time course of the ‘gliding mesh’ network with all microtubules shown in gray filaments (left) and a subset of microtubules colored distinctly (right). Total microtubule length per area is 10 μm/μm^2^

The state of the gliding mesh was separated from the aster and parallel bundle states by the extensile bundle state which was characterized by high values for the order parameter, cluster size, and sliding speeds.

**Figure 6:**
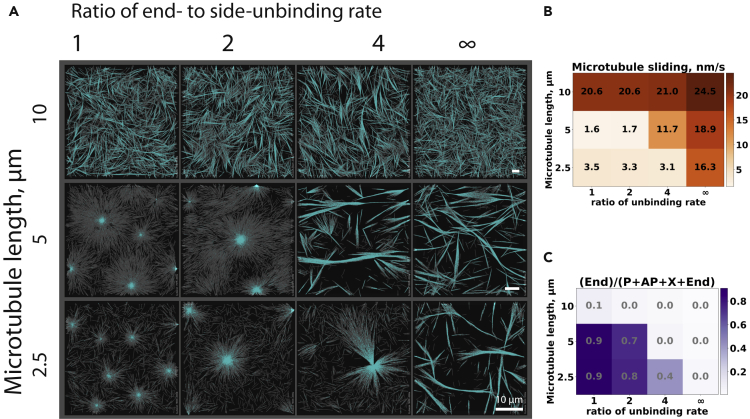
Organizational phase space and active network characteristics at various motor end-unbinding rates (A) Microtubule organizations with increasing motor end-unbinding rates for three different individual microtubule lengths at a constant total microtubule length per area of 5 μm/μm^2^. There are 16 KIF11 motors per microtubule. The simulation lasted for 60 min in a box of dimensions: Lx μm × Ly μm × 0.2 μm with periodic boundary conditions in x and y dimensions, where Lx = Ly = 16 × (microtubule length). All scale bars are 10 μm. (B) Calculated mobility of microtubule minus ends at 60 min in each simulation. (C) Fraction of end links for each simulation shown in (A). See also Video S4.

Because changing the microtubule length changes the ratio between microtubule end-bound and side-bound motors which in turn controls network organization, we asked whether the location of the boundary between aster and extensile states in the organizational phase space can be modified by changing the end-unbinding rate of the motors. We found that the network states characterized by high microtubule sliding speeds indeed occupied a larger area of the parameter space when the end-unbinding rate was increased (Figures 6A and 6B), corresponding to decreased end accumulation (Figure 6C). Aster formation was suppressed, and extensile bundling was promoted (Video S4). These observations indicate that microtubule length changes can, at least to a certain extent, be compensated for by kinetic rate changes in the motors.

Video S4. Time course of the organization of microtubules (gray) and KIF11 motors (cyan) in the absence of an attractive bundling force that form asters at low end-unbinding rate (left, density: 5 μm/μm^2^, L: 5 μm; ratio of end-unbinding rate to side-unbinding rate: 2) and extensile bundles (right, density: 5 μm/μm^2^, L: 5 μm; ratio of end-unbinding rate to side-unbinding rate: infinite)

### Experimental demonstration of the existence of the “gliding mesh” state

**Figure 7:**
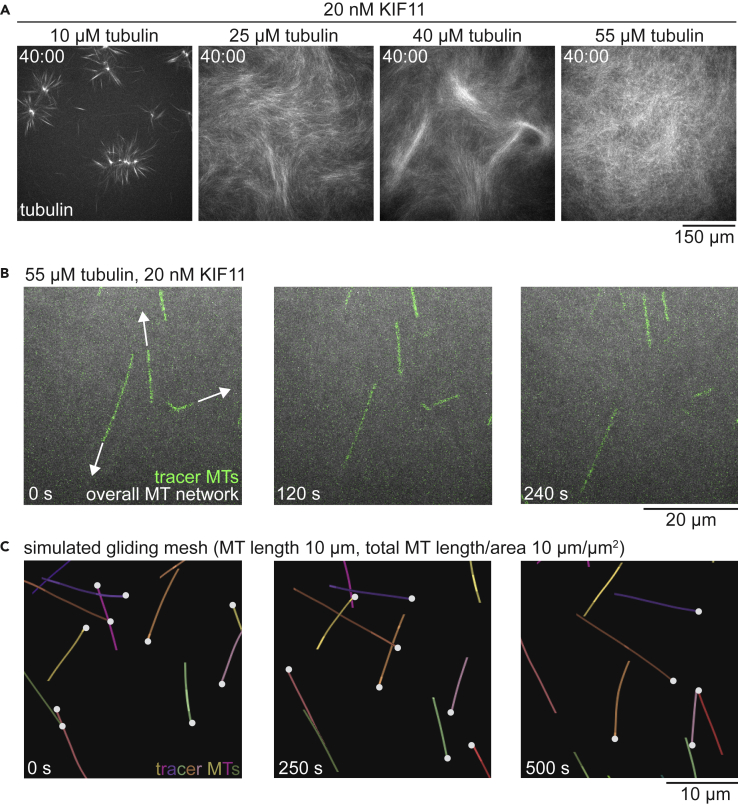
Experimental observation of the isotropic gliding mesh state (A) Confocal fluorescence microscopy images showing CF640R-labeled tubulin in microtubule networks polymerized and organized in the presence of 20 nM KIF11. With increasing tubulin concentration, the macroscopic network state transitions from contractile (10 μM tubulin) to active nematic (25, 40 μM tubulin) and to isotropic (55 μM tubulin). See also Video S5. (B) Time course at higher magnification showing fluorescence of AlexaFluor568-labeled GMPCPP tracer microtubules (green) as they glide through an overall isotropic network organized by 20 nM KIF11 from 55 μM tubulin (CF640R-tubulin; gray). Microtubules translocated through the network with an average speed of 32.4 nm/s (SE = 1.6; Figure S6). 0 s timepoint is 49 min after temperature shift. See also Video S6. (C) Simulated trajectories of tracer microtubules. A subset of microtubules is shown with the minus ends marked by circles. The total microtubule length per area is 10 μm/μm^2^, and the microtubule length is 10 μm (See also Video S3B).

Finally, to test if the “gliding mesh” predicted by our simulations existed or not, we performed experiments. Microtubules were nucleated from purified tubulin in a glass chamber and organized by purified KIF11 motor proteins (see STAR Methods). We varied the tubulin concentration and saw that KIF11 forms contractile networks of asters at low densities of microtubules and active nematic networks at higher microtubule densities (Figure 7A, Video S5), as shown previously.^15^^,^^16^ At the highest tubulin concentrations (where microtubule density and length are expected to be the highest), the network instead formed a cross-linked mesh without obvious macroscopic ordering, corresponding to the “gliding mesh” state found in the simulations.

Video S5. Time course of experimental self-organizing microtubule networks in 20 μm high chambers, driven by 20 nM KIF11, in the presence of 10, 25, 40, and 55 μM tubulin (CF640R-tubulin; final labeling ratio 3.5%); confocal images for each experiment are taken at the chamber midplane

Incorporation of pre-polymerized, stabilized tracer microtubules labeled with a different fluorophore showed that despite the lack of macroscopic order, individual microtubules were largely motile and translocated in the direction of their longitudinal axes (Figure 7B; Video S6), resembling microtubules in the corresponding simulations (Figure 7C). Microtubules translocated through the network with an average speed of 32.4 nm/s (SE = 1.6; Figure S6), close to the previously reported velocities of KIF11-driven microtubules on a surface^15^ and of single KIF11 molecules moving on immobilized microtubules.^22^ In the simulations, microtubules in the gliding mesh also approached the speed of the motor (Figure 4C). The characteristics of the experimental network and microtubule movements thus closely match the ones found in the simulation, and this confirms experimentally the existence of the predicted “gliding mesh”, a new type of active microtubule network.

Video S6. Translocation of pre-polymerized, stable GMPCPP tracer microtubules (14% AlexaFluor 568; left) in an overall isotropic network driven by 20 nM KIF11 (55 μM tubulin, 3.5% CF640R-tubulin; right) in a 20 μm thick chamber, demonstrating the 'gliding mesh' state

## Discussion

Here we studied two important control parameters of active microtubule network organization that have so far escaped substantial theoretical investigation: (i) short-range depletion forces between microtubules and (ii) the length of microtubules. In experiments, depletion forces can be manipulated by adding crowding agents. Microtubule length is more difficult to control *in vitro* because experimental parameters affecting microtubule length often also change microtubule density and growth speed, making the influence of only the microtubule length on network organization challenging to dissect from experiments alone. Living cells, however, have evolved many mechanisms to control microtubule length, as this is clearly a key parameter controlling the organization of microtubule networks.^23^^,^^24^

We were able to reproduce in our simulations of microtubule/motor networks the transition from asters to networks of extensile bundles by increasing the strength of short-range attraction between microtubules, mimicking the depletion effect produced by crowding agents in previous experiments.^14^ We found that the microtubule density needed to be high enough to allow constant remixing of the extensile microtubules bundles by fusion. This remixing is required to compensate for local microtubule polarity sorting. At lower microtubule densities, bundles are instead polarity sorted because they fail to fuse and remix, explaining why a certain microtubule density is required to achieve a network of permanently extensile bundles.^13^^,^^14^

Previous work showed that the transition from asters to nematic networks can also be obtained with dynamic microtubules and motors in the absence of crowding agents when the tubulin concentration was increased.^15^ The tubulin concentration affects the number of microtubules, their growth speed, and their length. Simulations explained that the increase in microtubule number and growth speed promoted nematic network formation by favoring microtubule side-to-side links over end links.^15^ However, the effect of the microtubule length remained unexplored. Here we find in our simulations that the formation of networks of extensile bundles is also promoted by increasing the microtubule length, essentially for the same reason, namely that side-to-side links become favored over end links.

But beyond a certain microtubule length and density threshold, a qualitatively new state emerges: the “gliding mesh”. Long microtubules cannot easily reorient as the network is highly connected. Instead, they continuously slide unidirectionally through the isotropic network with the motor speed, whereas the positions of the cross-linking motors are rather static. This microtubule sliding behavior in a highly percolated network agrees with a previous coarse-grained theory,^25^ and the gliding mesh state may already have been observed in previous experiments with high tubulin and high motor concentrations, where it was described as “unorganized” or “stuck”, as no attempt was made to visualize potential microtubule sliding in the dense isotropic network.^15^ Here we tested the prediction of the simulations experimentally and were indeed able to reproduce the transition from a contractile network forming asters to a network of extensile bundles and finally to the “gliding mesh” state by increasing the tubulin concentration. Microtubules sliding through the “gliding mesh” was observed directly by labeling a subset of the microtubules.

It is known from past simulations that the microtubule end-unbinding rate of the cross-linking motors needs to be slow enough to allow aster formation.^11^^,^^15^^,^^26^ We showed here that this control parameter shifts the boundaries between the different network states. As aster formation becomes more difficult with an increasing end-unbinding rate, the nematic network state becomes more accessible for short microtubules. Tuning this kinetic parameter in simulations can be useful from a practical point of view. Our simulations with the longer microtubules whose lengths can easily be reached in experiments are extremely time-consuming (∼2 weeks) because the simulation space had to be very large to avoid artifacts caused by the boundaries. The simulation space and time can however be reduced significantly using shorter microtubules and a higher end-unbinding rate to compensate for their stronger tendency to form asters. This then allows one to simulate the experimentally observed network transitions with shorter microtubules.^16^ It also suggests that living cells of different size need to modify not only microtubule length but also kinetic properties of associated proteins such as cross-linking motors in order to adjust the scale of a particular type of microtubule network.

In conclusion, we have used computer simulations to show that the microtubule length and the strength of a short-range attraction between microtubules that mimics a crowder-induced depletion force are important control parameters for the active network organization of microtubule/motor systems. Our simulations are in good agreement with previous experiments and have made a prediction regarding the dynamic state of a highly connected isotropic network that we could confirm experimentally. The simulations have the advantage of allowing the different topologically defined types of motor cross-links that characterize the network self-organizations to be extracted over the course of their development, which is currently not possible in experiments, and the behavior of these cross-link types can provide mechanistic insight into the principles that drive active network formation. Our results here expand our understanding of the effects that control parameters have on active microtubule networks, help to better understand the control of active network architectures in cells, and may also help to engineer novel biomimetic or bioinspired materials.

### Limitations of the study

The short-range attractive force in our model represents the “depletion force” generated by crowding agents. This weak force specifically promotes the formation of bundles of microtubules in which the microtubules are still able to slide relative to each other. It also promotes the cross-linking activity of molecular motors but does not prevent them from sliding microtubules along each other. Crowding agents promote the association of molecules in general, and they are therefore expected to increase the binding rate of molecular motors to microtubules, but we have not included this effect in our model. Crowding also increases the drag on all objects, which we have also not included in our model, in order to focus on the most interesting aspect: the bundling. Moreover, in our model, we approximate both the short-range repulsive interaction and the attractive potential of the depletion interaction by a harmonic potential, with a different stiffness parameter on each side of the equilibrium point. This accounts for the asymmetry between the stiff repulsive force and the soft attractive force but may not represent all detail of the more complex “true”, however, less-known potential.

## STAR★Methods

### Key resources table

**Table:** 

REAGENT or RESOURCE	SOURCE	IDENTIFIER
**Chemicals, peptides, and recombinant proteins**		

KIF11-mGFP	Purified according to [Roostalu et al.^15^]	Corresponding recombinant DNA: pJR303
Pig brain tubulin	Purified according to [Consolati et al.^27^]	N/A
Catalase	Sigma-Aldrich	Cat#: C40
Glucose Oxidase	Serva	Cat#: 22778.01
β-casein	Sigma-Aldrich	Cat#: C6905
Docetaxel	Sigma-Aldrich	Cat#: 01885
GMPCPP	Jena Bioscience	Cat#: NU-405S
CF™ 640R succinimidyl ester	Sigma-Aldrich	Cat#: SCJ4600044
Alexa Fluor™ 568 NHS Ester	Thermo Fisher	Cat#: A20003

**Recombinant DNA**		

pJR303 (pFastBac-StrepTagII-KIF11-A3G5-mGFP)	From [Roostalu et al.^15^]	N/A

**Software and algorithms**		

Cytosim	Nedelec and Foethke,^21^	https://gitlab.com/f-nedelec/cytosim
Scikit-learn 1.1.2	open source	https://scikit-learn.org
Python 3.9.10	open source	https://www.python.org/
Pandas 1.3.4	open source	https://pandas.pydata.org/

**Deposited data**		

Code for simulation and analysis	this paper	https://zenodo.org/record/7588572

**Other**		

PEG-passivated glass coverslips	Prepared according to [Consolati et al.^27^]	N/A
Double-sided tape (PET-based)	Nitto Denko	Cat#: 5601

### Resource availability

#### Lead contact

Further information and requests for resources and reagents should be directed to the lead contact, Thomas Surrey (thomas.surrey@crg.eu).

#### Materials availability

Plasmids used in this study are available upon request.

### Method details

#### Model

We simulated active networks consisting of microtubules and motors using Cytosim (https://gitlab.com/f-nedelec/cytosim). Our aim was to systematically investigate the effects of key parameters and monitor the system organization using a limited set of scalar quantities calculated automatically. The model is essentially as described earlier.^15^^,^^16^^,^^26^ The software, simulation, and analysis scripts are deposited at Zenodo (https://zenodo.org/record/7588572).

In brief, microtubules are modelled as diffusive, flexible filaments that repel each other via soft-core interactions. We model the interaction between microtubules using a piecewise linear force that is repulsive below range $d_{0}$, attractive between $d_{0}$ and $d_{1}$ and null above ${d_{0}+d}_{1}$:

$$F\left(d\right)=k\left(d-d_{0}\right),with\;k=\left\{ \begin{array}{ccc} K_{r} & if & d\leq d_{0}\\ K_{a} & if & d_{0}<d\leq d_{0}+d_{1}\\ 0 & if & d_{0}+d_{1}\leq d \end{array}\right.$$

where *d* is the distance between two interacting vertices of the filaments. This force is characterized by distances *d*_*0*_ and *d*_*1*_, and by *K*_*r*_ and *K*_*a*_ the repulsive and attractive force stiffnesses. In a bundle at equilibrium, the filaments are typically separated by *d*_*0*_, measured center-to-center. The force is orthogonal to the filament so as to permit sliding of the microtubules parallel to their axes.

The piecewise linear force approximates the steric interaction between microtubules by a soft-core repulsion, as done previously^21^ and approximates the depletion force generated by crowding agents by a short-range attraction. The depletion interaction between two hard cylinders has been calculated analytically using the Derjaguin approximation, but this approximation assumes that the separation is small compared to the radius of the cylinders, and this does not seem to be valid in the case of microtubules, which in a bundle are typically separated by a distance about equal to their diameter. The depletion force between cylinders in a fluid of hard spheres has been calculated numerically^28^^,^^29^ but it is unclear if the conditions considered by these authors match our experiments. The potential posited in our model can be seen as an approximation to what is unfortunately an unknown interaction potential, around its minimum. A linear force (quadratic potential) is simply the first nonzero term in the Taylor series.

The length scale of the depletion interaction depends on the size of the depletant. Polyethylene glycol (PEG) used in *in vitro* microtubule experiments typically ranges from 14-20 nm in diameter of gyration^14^^,^^30^^,^^31^ whereas the hardcore diameter of a microtubule is 25 nm. In our model we use a larger range for both the attractive and repulsive interaction: d_0_ = 0.1 μm and d_1_ = 0.32 μm. This allows bundles to form with lower density of filaments, which reduces the computational cost of the simulations, enabling us to simulate systems of the size as investigated here (individual simulations required at least 2 weeks of computation).

We mimic the concentration dependent depletion strength by varying K_a_ from 0.3 to 1.2 pN/μm. In our linear force approximation, the maximum mechanical work of the attractive force is approximately (*K*_*a*_)(*d*_*1*_)^2^ / 2=(1.2 pN/μm)(0.32 μm)^2^ / 2 = 0.061 pN μm = 14 kT which is well within the previously estimated range of 4-40 kT per micrometer of microtubule for the interaction energy between two parallel cytoskeletal filaments.^30^^,^^32^ The maximum repulsive energy in our model is (*K*_*r*_)(*d*_*0*_)^2^ / 2 = (50 pN/μm)(0.1 μm)^2^ / 2 = 0.25 pN μm = 60 kT, much larger than the attractive interaction.

Microtubules were introduced as “seeds” that nucleated at the very beginning of the simulation. A nucleated microtubule grows only at the plus end with a gradually reducing speed, mimicking the depletion of solubule tubulin, as in previous work.^16^ The time-dependent growth rate follows $v_{g}\left(t\right)=\alpha \left[1-\left\{\sum L_{i}\left(t\right)\right\}/\Omega \right]$ where α is the growth speed*,* $\sum L_{i}\left(t\right)$ is the total length of all microtubules at time *t,* and Ω is the available amount of tubulin subunits in the system, a parameter expressed in μm. By definition, the total length of all microtubules (some fraction of Ω) is the mean microtubule length multiplied by the number of microtubules. At a given microtubule density, we control the mean microtubule length by changing the number of microtubule*s* in the system*.*

Microtubule crosslinking motors can bind stochastically to two microtubules at most and walk along them in the plus-end direction. The properties of the motor are set to mimic those of human kinesin-5 (KIF11, also known as Eg5 in Xenopus), as in previous work.^15^^,^^16^ Microtubule bound motor can unbind with a higher rate at the end of microtubule than at the side. All simulations were performed in a flat and thin three-dimensional geometry with periodic boundary conditions in the X and Y dimensions and reflecting boundaries in the much shorter Z dimension, allowing the formation of extended quasi-two-dimensional networks.^15^^,^^16^^,^^26^ The size of the simulation box is Lx = Ly = 16L where L is the microtubule length. The thickness of the box Lz is 0.2 μm in all simulations presented here, and we thus quantify the system’s density by the total length of microtubules divided by Lx × Ly. Detailed parameters of the model are available in Table S1.

#### Experimental self-organization assay

Samples for the self-organization assays in Figure 7 were prepared similarly to previous work.^16^ Pig brain tubulin, recombinant KIF11-mGFP and tracer microtubules (GMPCCP seeds) were prepared as previously described.^15^^,^^16^^,^^27^ Passivated glass coverslips were prepared as described,^27^ however glass was cleaned by sonication in acetone followed by plasma cleaning in place of sonication in piranha solution. Chambers were prepared using two layers of 10 μm thick double-stick adhesive tape (Nitto Denko) for a final chamber height of about 20 μm. Tubulin (including CF640R-labeled tubulin at a final label ratio of 3.5%) and KIF11-mGFP (for reported concentrations referring to monomers) were mixed into an assay buffer on ice, centrifuged at 17,000 g at 4°C for 5 minutes in a table-top centrifuge, recovering the supernatant. The supernatant was transferred to a tube at room temperature, and mixed with BRB80 (80 mM PIPES, 1 mM MgCl_2_, 1 mM EGTA, pH 6.8) or GMPCCP tracer microtubules (AlexaFluor568; 14% labeling ratio) diluted in BRB80, for final assay component concentrations including 0.68 mg/mL glucose oxidase, 0.17 mg/mL catalase, 0.9 mg/mL β-casein, in a buffer of 40 mM PIPES, 1 mM EGTA, 1.6 mM MgCl_2_, 0.9 mM ATP, 0.58 mM GTP, 32 mM glucose, 3.2 mM β-mercaptoethanol, and 1 μM docetaxel, for a final pH of 6.9 – 6.95. Chambers were preheated to 33° C on a heat block and washed with BRB80 buffer just before loading the final sample and sealing with silicone vacuum grease. Imaging was performed as previously described^16^ on a spinning disk confocal microscope at 33° C around 3 minutes after the initial temperature shift, which stimulates microtubule nucleation and growth. Images were analyzed using Fiji.^33^ Timestamps refer to time since the beginning of imaging. Panels in Figure 7A are single slices from the chamber midplane, whereas panels in Figure 7B are maximum-intensity projections of 5 slices, 1 μm apart, around the chamber midplane. Intensities are adjusted independently for each experiment.

### Quantification and statistical analysis

#### Nematic order parameter

In dimensionality *d*, the orientational order is characterized by a symmetric traceless *d*×*d* tensor^34^^,^^35^ built using the outer product ⊗:

$$Q=\left\langle \hat{u}⊗\hat{u}\right\rangle -\frac{1}{d}$$

Where $\hat{u}$ is a unit *d*-vector directed along the axis of microtubules, and ⟨⋅⟩ denotes ensemble average over all microtubules. The scalar order parameter *S* is the largest eigenvalue of *Q.* Since our system is quasi-2D, we calculated a 2D order parameter, only considering the X and Y components of the filament’s 3D direction vectors, rescaled such that ${{\hat{u}}_{x}}^{2}+{{\hat{u}}_{y}}^{2}=1$. By construction, S∈[0,1]. If the system remains isotropic (no alignment), *S* is close to 0. If the alignment is perfect (nematic or vectorial), *S* is equal to 1.

To capture the local nematic order, the sampling window must be smaller than the simulation box, but this window size must be chosen carefully. To differentiate asters from nematic bundles, we adjusted the window size to contain the largest aster observed, that is 10 μm × 10 μm. This lowers the order calculated for an aster since microtubules from the aster radiate in all directions. A large window size also lowers the order calculated when several bundles of different orientations are present in the window. Nevertheless, for sparse and thick bundles, the order value remains close to 1 in this study. To avoid overcounting the contribution of isolated microtubules, the order parameter of each sampling window is weighted by the number of microtubules in that window. The weighted average of the local order parameter of all windows gives the nematic order parameter of the entire system, referred hereon simply as *S*.

#### Crosslink types

To characterize different types of connection made by motor between microtubules, we defined four types of crosslinks: P link, X link, AP link and end links as described before.^15^ P links connect parallel microtubules where the internal angle is smaller than 60 degrees; X links connect microtubules with an angle between 60 and 120 degrees. AP-links connect antiparallel microtubules with an angle between 120 and 180 degrees. End links are motors crosslinks bound close to one or two microtubule plus ends (within a distance of 10 nm). We present in our figures the proportion of end links, expressed as the fraction of all other links, i.e. end links/( P+AP+X+end links). The proportions of the various non-end links (P, AP, X links) are expressed as fractions of the sum of only the non-end links, i.e. divided by (P+AP+X links).

#### Microtubule mobility

To calculate the overall speed of microtubule motion, we extracted the positions of microtubule minus ends at regular time intervals Δt. We then calculated the displacement component parallel to the microtubule axis. Averaging this signed scalar displacement for all microtubules and dividing by Δt gave the overall averaged microtubule speed. A large Δt = 200 s was chosen to ensure that the contribution of diffusion becomes negligible relative to the active motion generated by the motors. As a matter of convention, a positive speed indicates minus-end leading sliding (as driven by plus-end directed motors).

#### Cluster size analysis

To quantify the connectivity of the microtubule network, we identify all clusters connected by motor crosslinkers. Then we calculate the size of the largest cluster, indicated by the number of microtubules in this cluster, and divide it by the total number of microtubules. When all the microtubules in the system are all interconnected, the size of the largest cluster will equal one.

#### Principal component and cluster analysis

We aim to categorize the simulated microtubule organization based on the seven descriptors of the network state, i.e. local order parameter, microtubule mobility, size of the largest cluster, fractions of P links, AP links, X links, and end links (fractions defined as in 'Crosslink Types' and as shown in Figures 3A and 5A) using a clustering algorithm. To reduce the dimension of the data set, we perform principal component analysis (PCA)^36^ on the seven descriptors using the Scikit-learn library (https://scikit-learn.org). In our analysis, the first two principal components can explain 94% of the variances where the loading vectors are tabulated in Figures S2A and S2C. We then categorize the microtubule network in this subspace using the K-means clustering method (N=4)^37^ to obtain the distinct clusters. To test the robustness of the PCA and cluster analysis (Figure S4), we repeated the analysis with all possible combinations of six descriptors only. We found that most of the identified clusters remain intact (see Figure S4), only a small number of networks near the boundaries of the clusters are a little less well defined.

#### Quantification of microtubule gliding speeds in experimental assay

15 minute Z-stack movies (21 steps of 1 μm, 1 min intervals) of tracer microtubules gliding in an isotropic network (described above; 55 μM tubulin, 20 nM KIF11, recorded 49 minutes after temperature shift) were projected onto a single slice by maximum intensity for each time point, and processed by background subtraction and median filtering to facilitate the identification of individual microtubules. Microtubules were then tracked manually using the TrackMate plugin for Fiji,^38^ selecting an arbitrary end of each microtubule as the tracked point. Total displacements were calculated for tracks longer than 7 frames to determine average speeds.

## Data Availability

• Cytosim files have been deposited at Zenodo and are publicly available as of the date of publication. The link is listed in the key resources table. Microscopy data reported in this paper will be shared by the lead contact upon request. • All original code has been deposited at Zenodo and is publicly available as of the date of publication. The link is listed in the key resources table. • Any additional information required to reanalyze the data reported in this paper is available from the lead contact upon request.
