# Supplementary material for: Effects of microtubule length and crowding on active microtubule network organization
Source: iScience. 2023 Jan 27;26(2):106063. doi: 10.1016/j.isci.2023.106063 (PMC9958361; doi:10.1016/j.isci.2023.106063)
Supplement: Document S1. Figures S1–S6 and Table S1 [file mmc1.pdf]

iScience, Volume *26*

Supplemental information

Effects of microtubule length and crowding on active microtubule network organization

Wei-Xiang Chew, Gil Henkin, François Nédélec, and Thomas Surrey

Figure S1. Size of the largest microtubule cluster in two differently percolated networks over time, related to Fig. 1.

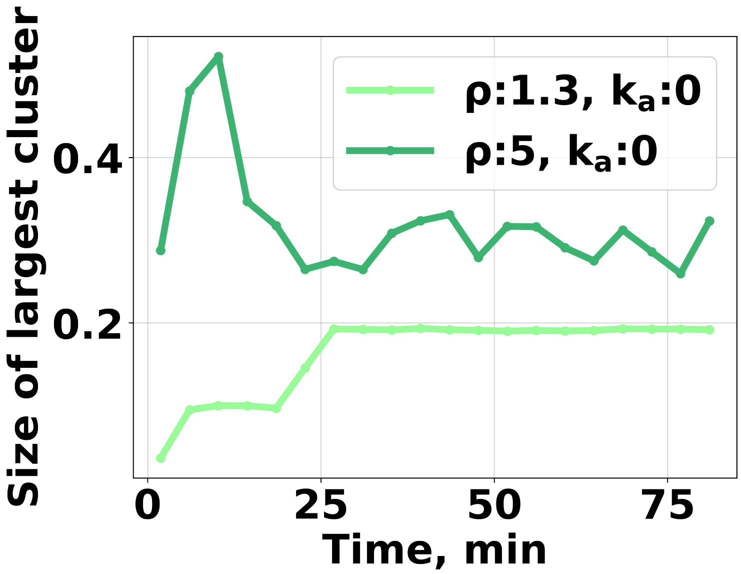

Figure S2. Details of PCA-cluster analysis and statistics, related to Fig.3 and Fig. 5.

A **Crowding**

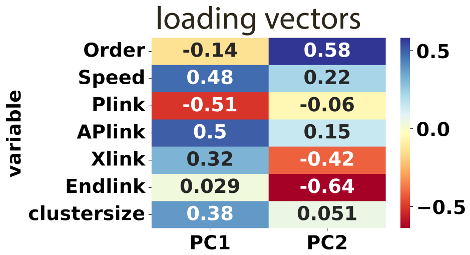

B

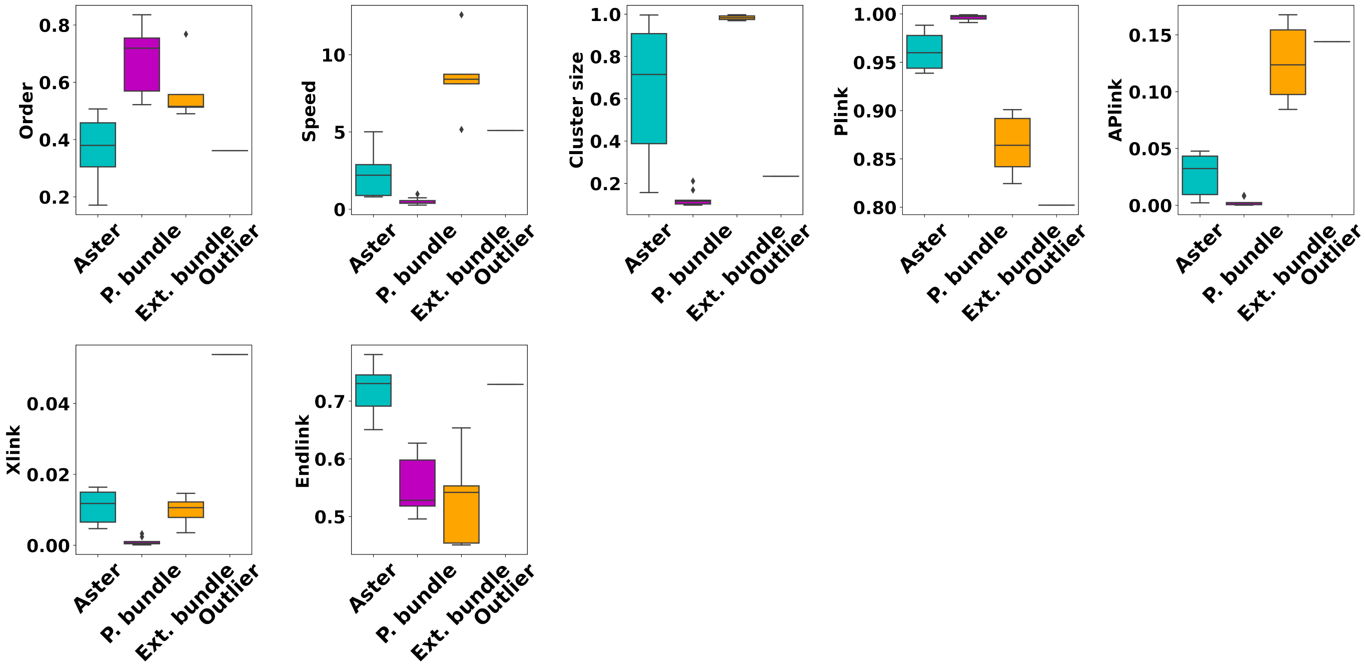

C **Long microtubule**

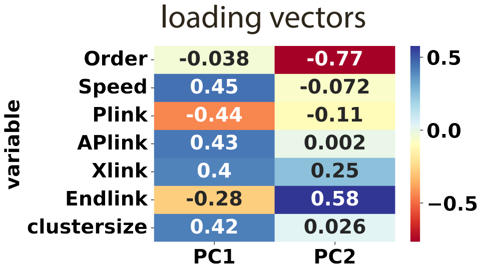

D

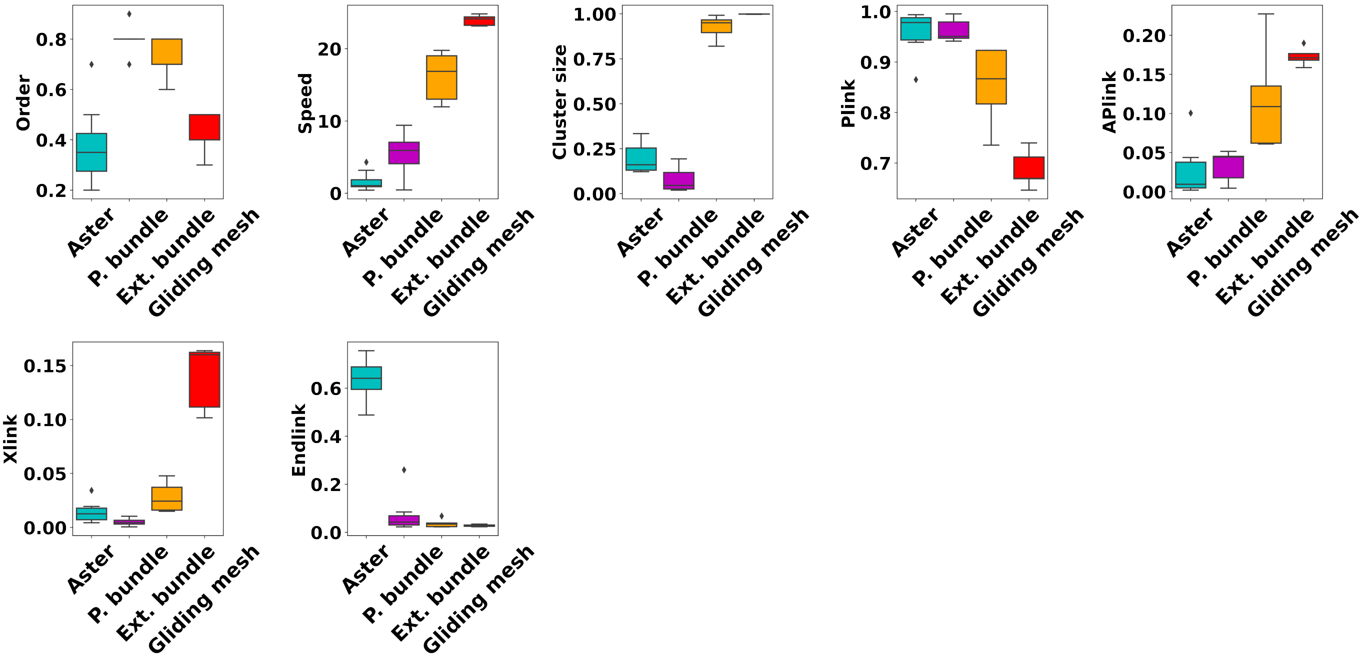

Figure S3. Organizational phase space of active microtubule networks at varying microtubule lengths displaying the entire simulated space, related to Fig. 4.

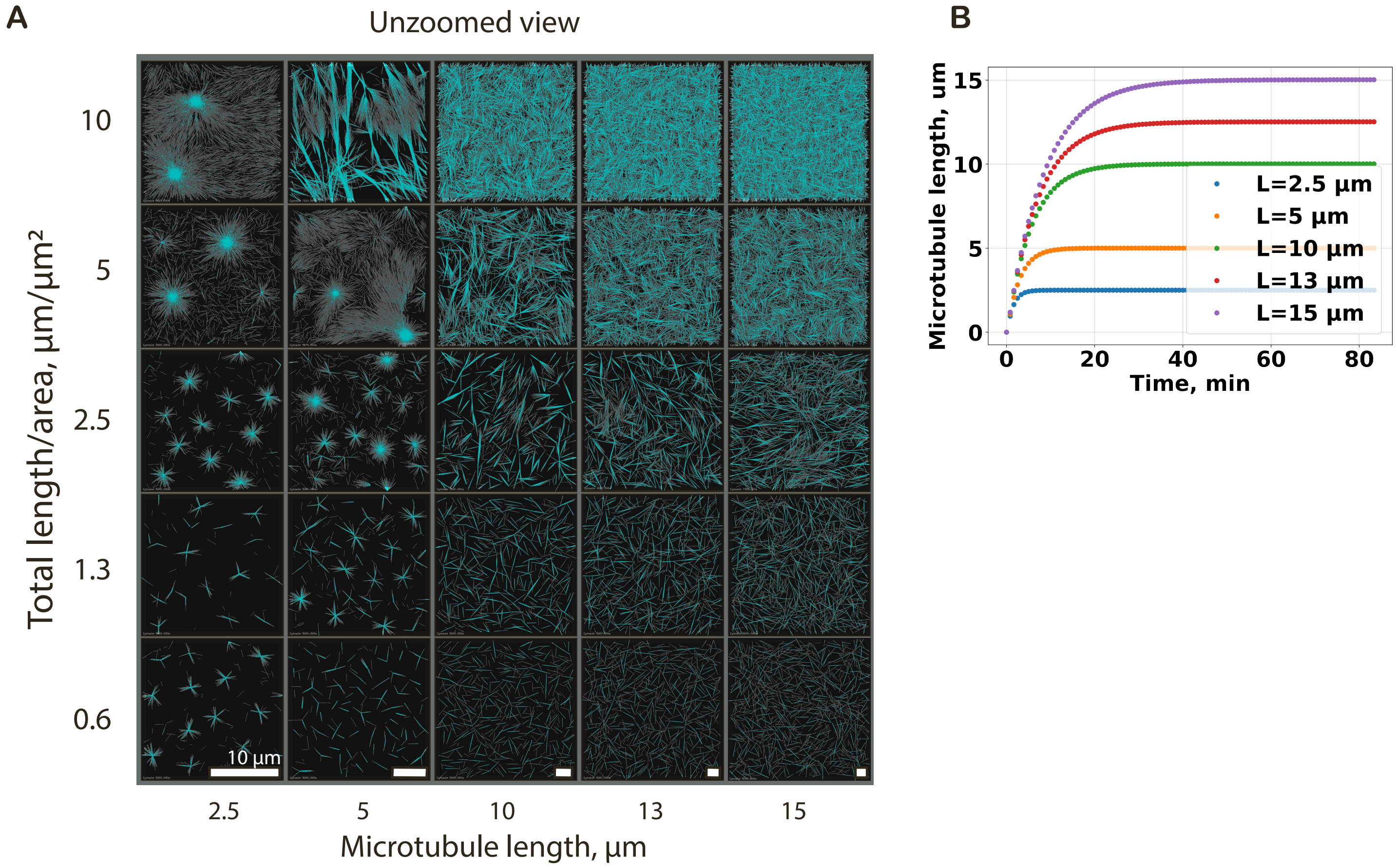

Figure S4. PCA-cluster analysis of networks formed at various microtubule lengths with one network descriptor excluded. Related to STAR Methods.

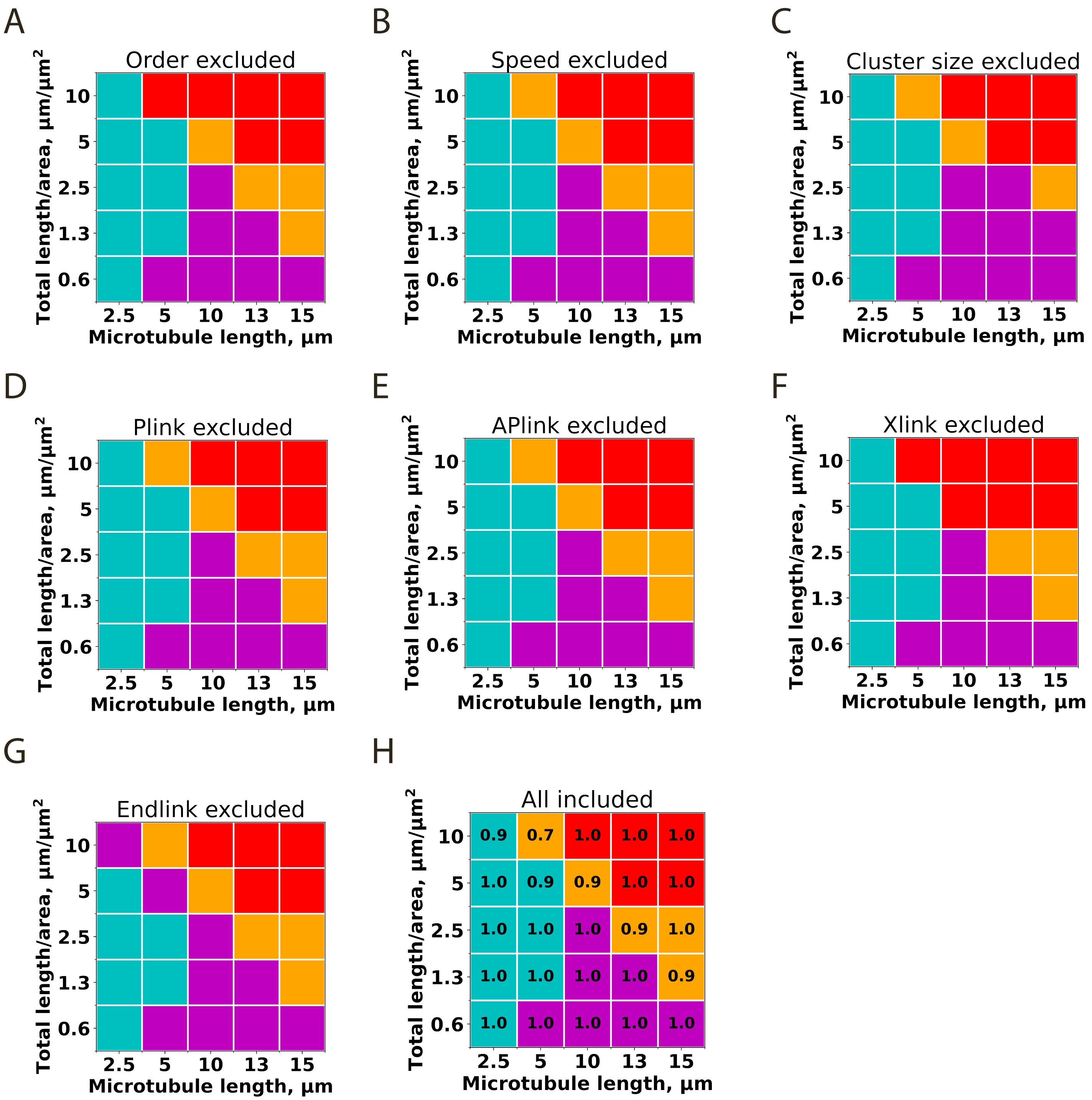

Figure S5. Topology of motor crosslinks with number values shown for the different crosslink ratios used for the PCA-cluster analysis, related to Fig. 3 and Fig. 5

Numerical version

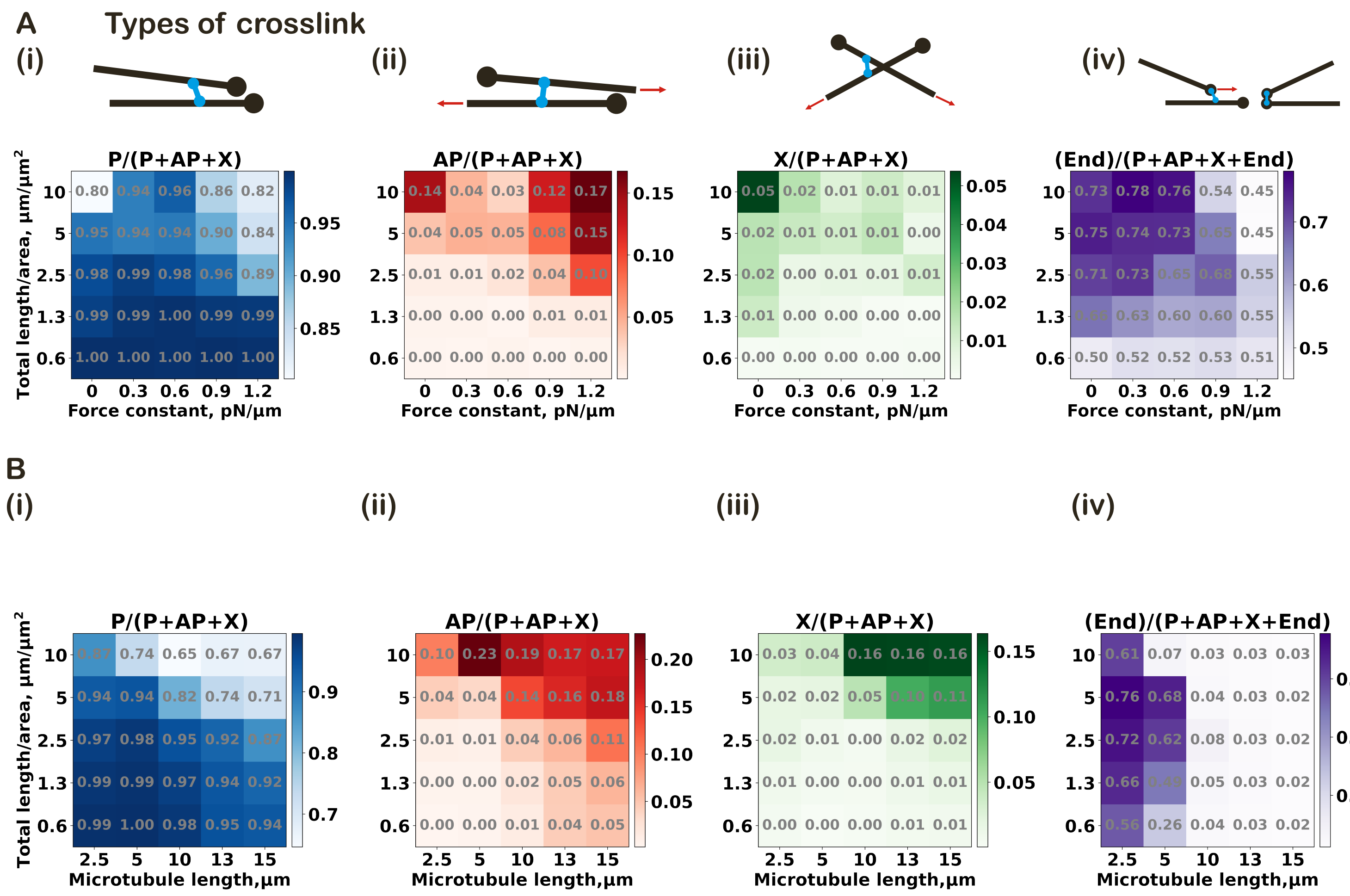

Figure S6. Histogram of tracked microtubule speeds in the experimental isotropic gliding mesh.

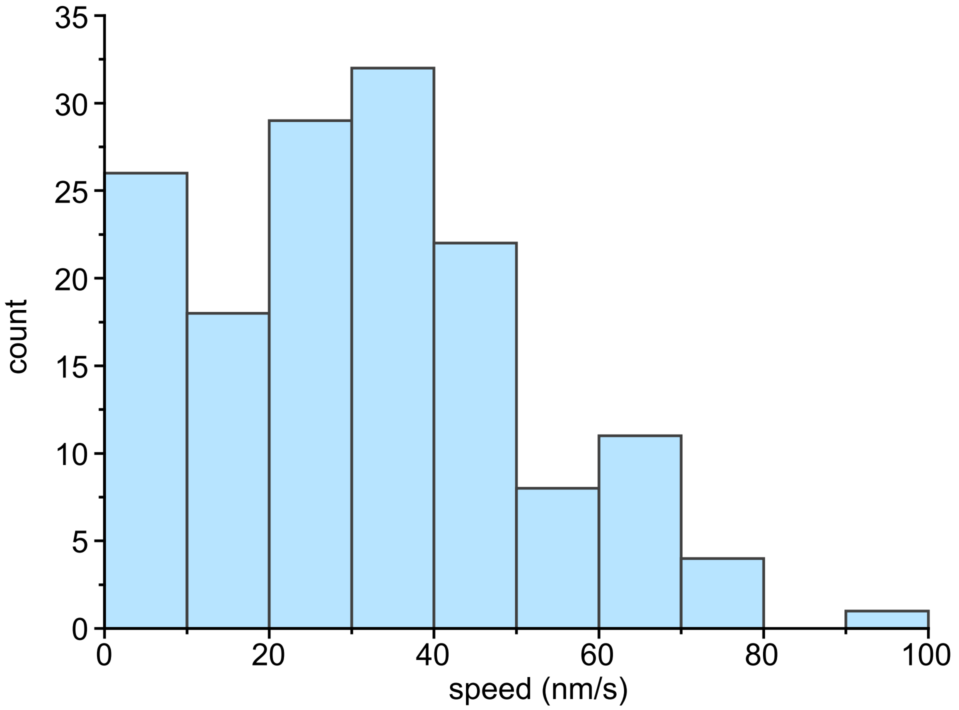

FIGURE LEGENDS

Figure S1. Time series of the size of the largest cluster for density: 1.3 μm/μm^2, k_a: 0 pN/μm and 5 μm/μm^2, k_a: 0 pN/μm (see Fig. 1C and 1D). The size of the cluster is normalized by the total number of microtubules in the system.

Figure S2. Breakdown of the loading vectors of each descriptors for the two principal components corresponding to analyses in (A) Fig. 3B and (C) Fig. 5B. Boxplot of each descriptors grouped by clusters corresponding to the analyses in (B) Fig. 3B and (D) Fig. 5B.

Figure S3. (A) Organizational phase space of the microtubule network at varying microtubule densities and lengths. The KIF11 motor to microtubule ratio is 16. The simulation was simulated for 60 minutes in a box of dimensions Lx μm × Ly μm × 0.2 μm, where Lx = Ly = 16 × (microtubule length). The full view of the simulation box is shown here. All scale bars are 10 μm. (B) Time course of microtubule mean lengths at different controlled lengths.

Figure S4. (A-G) Phase space of the different microtubule networks identified by PCA-cluster analysis with one of the network descriptors excluded, as indicated. (H) The phase space obtained when all descriptors are included in the analysis. The numbers indicate the probability of being classified as the indicated network in the analyses lacking one descriptor shown in A-G.

Figure S5. Numerical version of (A) Fig. 3A and (B) Fig. 5A.

Figure S6. Histogram of tracked microtubule speeds in experimental isotropic networks assembled from 55 μM tubulin and 20 nM KIF11 (see Methods). The histogram was constructed from 151 tracked microtubules across two experiments.

Table S1. Parameters used in the Cytosim computer simulations. Related to STAR Methods.

**Parameter**	**Value**	**Note**
**Simulation**		
Time step	0.01 s	Small enough for convergence
Viscosity	0.2 pN s/μm^2	200x of water
Box thickness	0.2 µm	Thick enough for 3-4 filaments to cross
**Microtubule**		
Length	2.5-15 µm	
Total length per area	0.6-20 µm/μm^2	
Rigidity	30 pN μm^2	Ref. [1]
Steric radius	0.05 µm	Ref. [2]
Repulsive force constant	50 pN /µm	Constrained by thermal fluctuations and force of motors
Attractive force constant	0.3-1.2 pN/µm	Same order of magnitude as experiment inferred depletion force [3,4].
Bundling force radius	0.16 µm	See Method
Growth rate	0.03 µm/s	Ref. [5].
Nucleation rate	0.1 /s	Time for all microtubules to nucleate is short relative to total simulation time
**KIF11 motor**		
Stall force	2 pN	Ref. [6]
Unbinding force	2 pN	Same order as in Ref [7]
Maximum motor speed	0.03 µm/s	Ref. [5]
Binding rate	0.5 /s	Estimated to match experiment
Unbinding rate	Side:0.1 /s<br>End: 0.2 /s	From run length =0.3 µm as measured in Ref. [8]
Binding radius	0.16 µm	1.5x crosslinker rest length
**Motor complex**		
Rest length	0.105 µm	Microtubule radius (0.025 µm) + actual crosslinker rest length (0.08 µm)[9]
Link stiffness	100 pN/µm	Ref. [10]
**Discretization parameter**		
Filament segment length	1 µm	Optimum for computation
Filament lattice size	8 nm	Size of a tubulin dimer
Motor stepping size	8 nm	Same as lattice size

References

1. Dogterom, M., and Yurke, B. (1997). Measurement of the force-velocity relation for growing microtubules. Science *278*, 856-860.
2. Nogales, E., Whittaker, M., Milligan, R.A., and Downing, K.H. (1999). High-resolution model of the microtubule. Cell *96*, 79-88.
3. Streichfuss, M., Erbs, F., Uhrig, K., Kurre, R., Clemen, A.E., Bohm, C.H., Haraszti, T., and Spatz, J.P. (2011). Measuring forces between two single actin filaments during bundle formation. Nano Lett *11*, 3676-3680. 10.1021/nl201630y.
4. Hilitski, F., Ward, A.R., Cajamarca, L., Hagan, M.F., Grason, G.M., and Dogic, Z. (2015). Measuring cohesion between macromolecular filaments one pair at a time: depletion-induced microtubule bundling. Phys Rev Lett *114*, 138102. 10.1103/PhysRevLett.114.138102.
5. Roostalu, J., Rickman, J., Thomas, C., Nédélec, F., and Surrey, T. (2018). Determinants of Polar versus Nematic Organization in Networks of Dynamic Microtubules and Mitotic Motors. Cell *175*, 796-808.e714. 10.1016/j.cell.2018.09.029.
6. Shimamoto, Y., Forth, S., and Kapoor, T.M. (2015). Measuring Pushing and Braking Forces Generated by Ensembles of Kinesin-5 Crosslinking Two Microtubules. Dev Cell *34*, 669-681. 10.1016/j.devcel.2015.08.017.
7. Andreasson, J.O., Milic, B., Chen, G.Y., Guydosh, N.R., Hancock, W.O., and Block, S.M. (2015). Examining kinesin processivity within a general gating framework. Elife *4*. 10.7554/eLife.07403.
8. Kapitein, L.C., Peterman, E.J., Kwok, B.H., Kim, J.H., Kapoor, T.M., and Schmidt, C.F. (2005). The bipolar mitotic kinesin Eg5 moves on both microtubules that it crosslinks. Nature *435*, 114-118. 10.1038/nature03503.
9. Kashina, A.S., Baskin, R.J., Cole, D.G., Wedaman, K.P., Saxton, W.M., and Scholey, J.M. (1996). A bipolar kinesin. Nature *379*, 270-272. 10.1038/379270a0.
10. Loughlin, R., Heald, R., and Nedelec, F. (2010). A computational model predicts Xenopus meiotic spindle organization. J Cell Biol *191*, 1239-1249. 10.1083/jcb.201006076.
